# Adverse influence of multilevel socioeconomic status on physical activity: results from a national survey in Vietnam

**DOI:** 10.1186/s12889-020-08695-5

**Published:** 2020-04-25

**Authors:** Thi Hoang Lan Vu, Thi Tu Quyen Bui, Thi Kim Ngan Nguyen, Van Minh Hoang

**Affiliations:** 1 Department of Epidemiology, Hanoi University of Public Health, No. 1A Duc Thang Ward, North Tu Liem, Ha Noi, Viet Nam; 2 Department of Biostatistics, Hanoi University of Public Health, No. 1A Duc Thang Ward, North Tu Liem, Ha Noi, Viet Nam; 3 Hanoi University of Public Health, No. 1A Duc Thang Ward, North Tu Liem, Ha Noi, Viet Nam

**Keywords:** Physical activity, Socioeconomic, Multilevel, Vietnam

## Abstract

**Background:**

This study aims to explore associations of individual- and provincial-level socioeconomic status (SES) and the combined interaction among these SES with individual physical activity (PA).

**Method:**

This analyze used data of 3068 Vietnamese people aged 18–65 years from the national representative STEPS survey in 2015 (STEPS2015). The survey collected PA-related data using the Global PA Questionnaire Version 2 and those on provicial-level characteristics from two surveys in 2014, namely the Intercensal Population and Housing Survey (IPHS) and The Vietnam Household Living Standard Survey (VLSS2014). Multilevel linear analyze was performed with individual and provincial characteristics as independent variables and the metabolic equivalent (MET) score – the indicator of individual PA – as the dependent variable.

**Results:**

Male and female participants with insufficient PA accounted for 20.2 and 35.7%, respectively. Both individual- and provicial-level SES were inversely associated with the individual PA level. As the provincial-level monthly income increased by 1 million Vietnam Dongs, the total PA score of individuals residing in that province reduced by 1900 METS. A buffering effect was reported between provincial and individual SES, as the provincial average income increased, the differences in PA scores between different SES groups decreased.

**Conclusion:**

Our data suggest that Vietnamese individuals in low SES groups tended to be more physically active than those in high SES groups because their PA was largely related to work.

## Background

Physical activity (PA) has long been well documented in the literature to have important health benefits, for example strengthening bones and muscles, or reducing risks and morbidity associated with non-communicable diseases (NCDs) [1–3]. Previous studies showed that PA benefits not only physical health but also mental health as it helps reduce symptoms of anxiety or depression [4]. Evidence on health benefits of PA has been available since the 1950s; however, the proportion of the population achieving sufficient PA remains low. According to the WHO’s data in 2010, about 23% of adults aged 18 years and over were insufficiently active. Besides, the figure in high-income countries was higher than that in low-income ones [5]. Physical inactivity is among of the most important public health problems of the twenty-first century because it is responsible for 6–10% of the major NCDs and 9% of premature mortality globally [1].

Socio-economic status (SES) is a key determinant of health and well-being because it can shape a person’s attitudes, practices and exposure to certain health risk factors [6]. When it comes to PA, social advantages allow for a higher PA level [7], and more importantly, the individual PA level was even affected by the SES of his or her residential area [8].

Neighborhoods have long been recognized as the structural conditions that shape individual lives and opportunities [9]. However, in public health, the role of physical and social environments in determining individual health behaviors remains controversial [10, 11]. Indeed, the contextual effects of neighbourhood on individual activities were mostly investigated in developed countries, and the definitions of neighbourhood varied across studies. For example, previous studies in Vietnam defined neighborhoods as administrative division such as provinces or districts. In our present study, we employed a multilevel design to explore how individual PA iss affected by individual- and provincial -level SES separately and the combined interaction among SES at different levels.

## Methods

### Data sources

Individual-level data: the STEPS survey 2015 was a cross-sectional survey applying the methods and tools of the WHO STEPS. The WHO Stepwise approach to surveillance (STEPS) is a simple, standardized method for collecting, analyzing and disseminating data in the World Health Organization (WHO) member countries [12]. The survey was conducted in all 63 provinces and cities of Vietnam, between June and October 2015.

Provincial-level data: data on provincial characteristics were extracted from two surveys: (1) The 2014 Intercensal Population and Housing Survey (a large sample survey implemented in the middle of two Population and Housing Censuses) and (2) The VLSS 2014.

Individual and provincial data files were linked together using the unique provincial identities (ID) in the 2009 Vietnam Population and Housing Census.

### Sample size

The sample size was calculated using the WHO’s sample calculator for STEPS. The samples were stratified by gender and three age groups (18–29, 30–49, and 50–69). The minimum sample size for each stratum was calculated according to the following formula:

$$ \mathrm{n}={Z}^2\frac{P\left(1-P\right)}{e^2} $$

In which z = 1.96, e = 0.05, *p* = 0.5. Then considering design effect of 1.5, an expected response rate (80%), and the number of stratum age/sex groups (6), the total sample estimated was 4320. With the response rate of 79.5% for all three STEP rounds, the final sample consisted of 3068 people aged 18–69 years old residing in Vietnam at the survey time.

### Sampling method

A 2-stage random systematic sampling method was used. The sampling frame for the survey was developed by the General Statistics Office of Vietnam (GSO) based on the master sampling frame used in the Population and Housing Census 2009 and updated with the 2014 data [13]. Based on the Population and Housing Census data 2009, the GSO used 15% of the master sample as the future national sampling frame for the STEPs survey. The master sample contains 25,500 enumeration areas (EAs) from 706/708 districts of Vietnam (2 island districts were excluded from the GSO master sample frame). Besides, we employed a selection probability proportional to size (PPS) sampling method, where the size calculated by dividing the selection probability of an EA from the entire target population by the selection probability of an EA from the master sample. The master sample frame of GSO was stratified by two variables: urbanization (1 = urban; 2 = rural) and district group (1 = district/town/city of province; 2 = plain and coastal district; 3 = mountainous, island district). In other words, it consisted of 6 sample frames (or strata). In the first stage of the sampling process, 158 EAs in the urban areas were selected for the survey, equating the number of those in the rural areas.

The second stage of sampling involved the selection of 10% of households in each EA. Thus, 15 and 14 households were respectively selected from these urban and rural EA, using a simple systematic random sampling method. Finally, STEPS 2015 recruited 4651 households, each of which had one eligible member automatically selected for the STEP 1 interview by the PDA program. The probability of selection was the product of the probabilities of selection for each stage. The sampling base weight of an eligible individual was the inverse of this selection probability.

### Measurement

#### Study outcomes

Total MET score: In the STEP survey in 2015, PA-related data were collected using the Global PA Questionnaire Version 2 (GPAQ-2) [14]. The GPAQ-2 contains 16 questions on frequency (days) and duration (minutes/hours) of moderate and vigorous-intensity PA in three domains for a typical week, namely work, transport, and recreation. The data were then converted into energy expenditure measured by Metabolic Equivalent Tasks (METS, a ratio between the working metabolic rate and the resting metabolic rate) following the instruction in the GPAQ-2 [14]. The total MET score is the sum of all METS/minutes/week from moderate-to vigorous-intensity PA in three domains.

Not meeting the WHO’s PA recommendations: According to the WHO, “throughout a week, including activity for work, during transport and leisure time, adults should do at least an equivalent combination of moderate- and vigorous-intensity PA achieving at least 600 MET-minutes”. Therefore, a person with a total MET score of < 600 MET-minutes in this study was defined as “not meeting the WHO’s PA recommendations”.

#### Independent variables

##### Subject-specific characteristics (level 1)

The following demographic variables of the survey participants were evaluated: 1) gender (i.e., male/female); 2) age group (i.e., 18–29; 30–49; and 50–69 years old); 3) educational level (i.e., less than primary education, primary school, middle school, high school and university/college and higher); 4) current primary occupation (i.e., farmer, government staff and others, including housewives, small traders, temporary workers, housekeepers, handicraft makers and jobless people, etc.); 5) type of residential area (i.e., urban and rural); and 6) household economic status. We measured household economic status based on an asset-based wealth index, constructed them using principal component analysis (PCA), and divided them into quintiles (i.e., lowest, lower middle, middle, upper middle, and highest). Previous studies had suggested the use of PCA to create a proxy indicator of SES from the above items in the Vietnamese context [15].

##### Province-specific characteristics (level 2)

The socio-economic characteristics of 63 provinces were measured using two variables: (1) *Provincial monthly income* per capita*:* the average per capita income from all sources in 2014; and (2) *Provincial urbanization rate:* the percentage of the population living in urban areas in the province in 2014. That information was extracted from the VLSS 2014 [16].

### Statistical approach

This study employed the approach to multilevel data analysis developed by Bryk and Raudenbush’s [17]. This approach enabled us to collect data on the direct effects of individual and provincial-level factors on the total MET score and adjusted standard errors due to the effects of clustering of subject-specific measures within provinces. We designed three models as follows: 1) the first model or empty model had with no explanatory variables; 2) the second model included all individual-level variables; and 3) the third model examined variables at both individual and provincial levels. The data were extracted from the census with survey weight. We used MLwiN to analyze both scaled weighted data and unweighted data, however, weighted and unweighted data did not diverge significantly in general. To examine the contribution of provincial factors to individual total PA score, a null linear model (without any individual or school factors) was fitted, in which the provincial variance was statistically significant and accounted for 19.5% of the total variability in individual total PA scores. Adding all individual variables to the model helped to reduce the variance at the provincial level to 13.9%. In the model incorporating all individual and provincial factors (presented in Table 3), the variance at the school level still stayed significant but now only accounted for 11.8% of the variability in the outcome.

## Results

### Characteristics of survey sample

Table 1 shows that 3068 out of 3856 individuals completed all three STEP rounds, giving a response rate of 79.5%. Females slightly outnumbered males (57.2% vs. 42.8%), and people aged 18–29, 30–49, and 50–69 years accounted for 15.9, 48.8, and 46.3%, correspondingly. About 62% of respondents reported being self-employed, and 68.5% had lower than secondary school education. An overwhelming proportion of the respondents belong to the Kinh group (80.2%). The highest and lowest income groups each formed 20.0%.

**Table 1:** General characteristics of the study respondents, STEPS Vietnam 2015

	Male 1314 (42.8%)	Female 1754 (57.2%)	Total n (%)
**Age group**			
18–29	219 (16.7)	268 (15.3)	487 (15.9)
30–49	610 (46.4)	887 (50.60	1497 (48.8)
50–69	485 (36.9)	599 (34.2)	1084 (35.3)
**Residence**			
Urban	572 (43.5)	803 (45.8)	1375 (44.8)
Rural	742 (56.5)	951 (54.2)	1693 (55.2)
**Ethnicity**			
Kinh	1066 (81.1)	1443 (82.3)	2514 (80.2)
Others	248 (18.9)	311 (17.7)	566 (19.8)
**Education**			
Primary school or less	196 (14.9)	368 (21.0)	564 (18.4)
Lower secondary	655 (49.8)	883 (50.3)	1538 (50.1)
Upper secondary	245 (18.6)	228 (13.0)	473 (15.4)
University/college	217 (16.5)	275 (15.7)	492 (16.0)
**Occupation**			
Government official	119 (9.1)	158 (9.0)	277 (9.0)
Non-government employee	139 (10.6)	145 (8.3)	284 (9.3)
Self-employed	873 (66.4)	1038 (59.2)	1911 (62.3)
Student	20 (1.5)	28 (1.6)	48 (1.6)
Homemaker	9 (0.7)	248 (14.1)	257 (8.4)
Retired	76 (5.8)	79 (4.5)	155 (5.1)
Unemployment	78 (5.9)	58 (3.3)	136 (4.4)
**Household economic status**			
Lowest	252 (19.2)	361 (20.6)	613 (20.0)
Lower Middle	334 (25.4)	468 (26.7)	802 (26.1)
Middle	191 (14.5)	237 (13.5)	428 (14.0)
Upper Middle	267 (20.3)	343 (19.6)	610 (19.9)
Highest	270 (20.5)	345 (19.7)	615 (20.0)

### Current situation of population physical activity

According to Tables 2, 28.1% of respondents did not meet the WHO’s PA recommendations, i.e. the total PA score of < 600 MET-minutes/ week (95%CI: 25.9 to 30.2%). Females had a significantly lower level of PA than males. The proportion of having no recreation-related PA was extremely high in all participants (70.6%) and females alone (74.7%).

**Table 2:** Situational analysis of physical activity among adults age 18–69-year-old in Vietnam, 2015

	Male		Female		Both gender	
	%	95% CI	%	95% CI	%	95% CI
Percentage of respondents not meeting WHO recommendations on PA for health	20.2	17.8–22.6	35.7	32.7–38.7	28.1	25.9–30.2
No work-related PA	39.8	36.7–43.0	56.2	52.7–59.6	48.1	45.6–50.7
No transport-related PA	64.2	60.8–67.7	50.5	47.2–53.8	57.3	54.6–59.9
No recreation-related PA	66.2	63.1–69.4	74.7	72.3–77.2	70.6	68.5–72.6

**Fig. 1:**
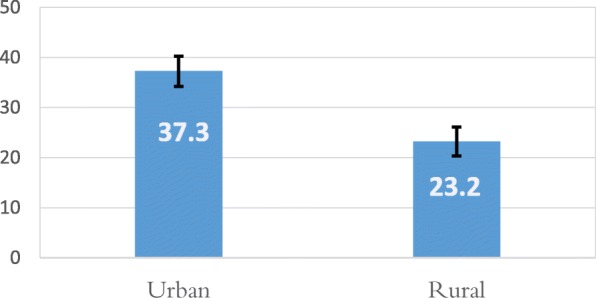
Percentage of respondents not meeting WHO recommendations on physical activity for health by rural/urban. Error bars represent the 95% confidence intervals (CIs) of the percentage estimated with generalized least squares

Figure 1 presents the proportion of respondents not meeting the WHO’s PA recommendations, by type of residential area. The proportion in urban areas (37.3, 95%CI: 34.4–40.3%) was much higher than that in rural areas (23.2, 95%CI: 20.3–26.1%).

### Inequities in physical activity levels across provinces

**Fig. 2:**
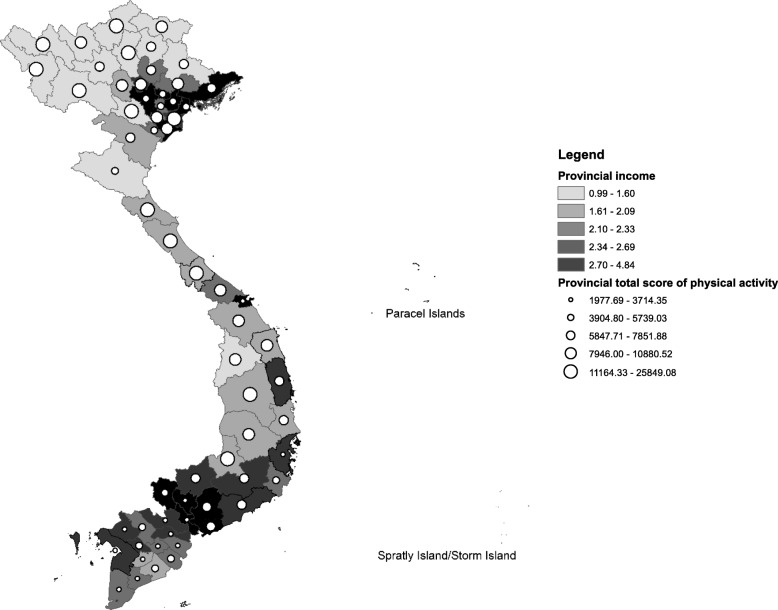
Negative correlation between Provincial average physical activity score and provincial average monthly income. This map presents the correlation between the provincial average MET score and the provincial average monthly income illustrated by the shade of the polygon. The darker the shade indicates a lower income, and the size of the dots presents the mean MET score, with bigger dots indicating higher PA levels. ***(****The map was created by the authors using ArcGIS software, with Vietnam administrative boundary shapefile and data extracted from STEPs survey and the VLSS 2014)*

The provincial average monthly income and the average provincial MET score varied significantly across 63 provinces in Vietnam. A negative correlation between these two factors was reported. The provinces with the lowest income per capita would be the ones with higher levels of physical activities (Fig. 2).

### Multilevel model for social determinants of total MET score

The multilevel model showed that both individual- and provincial-level factors had independent impacts on the individual-level total MET score. At the level 1, all demographic and socioeconomic factors were significantly associated with total MET score (Table 3). For instance, the 30–49 year-olds had a higher total MET score than the 18–29 year-olds (*p* < 0.05), and females scored higher than males (*p* < 0.05). Besides, individuals with better education/ SES tended to have lower total MET scores.

**Table 3:** Multilevel for social - determinants of total PA scores on METS

		***Null model***	***Model 1*** ***Coefficients (CI95%)***	***Model 2 Coefficients (CI 95%)***
**Fixed effects**				
***Individual level***				
***Demographic factors***	*Age 18–29*		Ref	*Ref*
	*Age 30–49*		526.5 (−0.74; 1053.7)	*573 (46.1; 1099.8)*
	Age 50–69		− 303.1 (− 886.2; 280)	− 253.1 (− 835.8; 329.6)
	***Female*vs.*Male***		− 1514.2 (− 1874.4; − 1153.9)	*− 1504.6 (− 1864.1; − 1145.1)*
	***Kinh*vs.*other ethnics***		− 1414.8 (− 2027.5; − 802.1)	*− 1104.1 (− 1733.6; − 474.5)*
***Socio-economic factors***	***Education***			
	Primary or less		Ref	Ref
	Lower secondary		63.4 (− 461.9; 588.7)	−45.0 (− 569.1; 479.1)
	*Upper secondary*		− 851.2 (− 1553.5; − 148.9)	*− 858.8 (− 1559.7; 157.9)*
	*College or above*		− 971.4 (− 1766.6; − 176.2)	*−973.6 (− 1767.6; − 179.6)*
	***Occupation***			
	Government employee		Ref	Ref
	Non-government employee		981.5 (130.3; 1832.7)	1024.6 (173.0; 1876.2)
	*Self-employed*		2411.2 (1677.6; 3144.8)	*2384.3 (1651.4; 3117.1)*
	Student		−409.0 (− 2030.1; 1212.1)	− 403.3 (− 2024.2; 1217.6)
	Homemaker		−973.9 (− 1909.8; −38.0)	− 974.1 (− 1909.0; −39.2)
	Retired		− 557.6 (− 1581.1; 465.9)	− 564.3 (− 1586.8; 458.2)
	*Unemployment*		− 2803.3 (− 3895.6; − 1711.0)	*− 2708.4 (− 3799.7; − 1617.1)*
	*Rural* vs. *Urban*		694.3 (291.3; 1097.3)	*673.7 (268.8; 1078.6)*
	***Household economic status***			
	Lowest		Ref	*Ref*
	*Lower Middle*		− 552.0 (− 1107.5; 3.5)	*− 3149.6 (− 5000.4; − 1298.8)*
	*Middle*		− 1308.5 (− 1966.9; − 650.1)	*− 3934 (− 6090.6; − 1777.4)*
	*Upper Middle*		− 1492.6 (− 2141.4; − 843.8)	*− 4766.1 (− 6777.3; − 2754.9)*
	*Highest*		− 1963.4 (− 2667.6; − 1259.2)	*− 5299.9 (− 7377.1; − 3222.7)*
***Provincial-level***				
Provincial urbanization rate in 2014				−13 (− 51.2; 25.2)
*Provincial average income* per capita*l in 2014*				*−1.9 (−3.1; −0.7)*
***Interaction between individual SES and provincial average income per capita***				
*Between middle low and average income*				*1.3 (0.5; 2.1)*
*Between middle and average income*				*1.3 (0.3; 2.3)*
*Between middle high and average income*				*1.6 (0.8; 2.4)*
*Between high and average income*				*1.6 (0.8; 2.4)*
**Random effects**				
*Province (variance)*		6,712,211	3,612,203	2,974,521
*Individual (variance)*		27,710,766	22,321,687	22,245,934
*ICC (%)*		19.5	13.93	11.79
*Change in -2loglikelihood*			691 (df = 19)	21 (df = 5)

At level 2, after controlling for all individual-level socioeconomic factors, the provincial-level average monthly income in 2014 had a negative association with the individual total MET score (*r* = − 1.9). Particularly, as this income increased by one million dongs, the total MET score of an individual living in the province reduced by 1900 METS. A cross-level interaction was found between the individual-level SES and the provincial-level monthly income.

## Discussion

This study used the data from a national representative sample of the Vietnamese population aged 16–69 years. We found that a high proportion of respondents did not meeting WHO’s PA recommendations; this result is quite consistent with those in previous studies [18–23]. Besides, respondents who did not engage in recreation-related PA accounted for the highest proportion, outnumbering those doing PA related to transportation or work. This can be explained by considering that most Vietnamese working-aged adults spend their time mainly on working, and therefore their duration of work-related PA was found longest [21, 22, 24].

This study employed a multilevel design to explore the independent impacts of individual- and provincial-level SES independently and the combined interaction among SES at different levels on individual PA. It was previously confirmed that people with higher SES tend to have better health and better lifestyles, including higher PA levels [25, 26]. There have been reports on positive impact of the contextual SES impact on PA, as residence in a deprived neighbourhood is often associated with lower PA levels [27]. This study, however, revealed a gradient in total MET scores across all SES measures at both individual and provincial levels. In particular, individuals with higher SES tend to have lower mean PA levels. Previous studies mentioned that the association between SES and PA, whether negative or positive, largely depends on the PA domains of interest. High SES groups were more likely to do PA during leisure time whereas those with low SES tend to be involved in occupational physical activity [28, 29]. Our survey results also showed a similar trend. Indeed, more PA done by our Vietnamese study participants occurred during work and transportation than during leisure time. This contributed to explaining the adverse association between SES and PA reported in our study and the higher level of PA in low SES groups.

More interestingly, a cross-level interaction between individual and provincial SES was reported. The positive coefficients of all the interaction terms demonstrated the buffering effect of the provincial average income on the adverse impact of individual SES on total PA scores. More specifically, as the provincial average income increased, the differences in PA scores between different SES groups decreased or the adverse impact of individual SES on PA was mitigated by the provincial SES. This can be explained by the higher availability of PA resources and their higher accessibility to wealthy people in these provinces [30].

In our survey, male adults undertook more PA than females. This result has been concluded by many other authors [18–22, 24, 31, 32]. Females were less likely to participate in either vigorous-intensity (occupational) PA or sports clubs than males [33]. Vietnamese society places household responsibilities on women; hence, they devote their time to doing household chores and taking care of children while spending less time on sports activities than men do. Besides, male adults are more likely to take part in transport-related PA such as walking and cycling because they are more active and have more comfortable clothing [31].

Our findings, like those of many other studies [20, 21, 34], show that urban dwellers were less involved in PA than those living in rural areas. A possible explanation for this is that people living in urban areas are more likely to work in offices and therefore adopt a sedentary lifestyle. Rural residents were more likely to be involved in labor-intensive jobs (e.g. farming or fishing) that require more vigorous-intensity PA. As a result, they had a higher total MET score than urban dwellers.

Like some other studies [21, 24, 28, 34, 35], our study pound that individuals with higher levels of education tended to participate in more PA than those with lower levels of education. The former had jobs that require mental labor or worked as white-collar workers Another finding of our study is similar to Stalsberg’s [28] in that both indicate that free workers (often, but not necessarily, having lower education), were largely blue-collar workers and those with comparatively low skill levels. They might be more involved in PA than government employees. In certain studies, better-educated individuals were more likely to engage in exercise/ sports during leisure time than those with lower educational achievement. However, their daily tasks at work tended to be more sedentary. Because they mainly participated in work-related PA (as mentioned above), their total MET score was lower than those with lower education levels. Therefore, education is a factor that helps in assessing the duration of each individual PA domain (such as leisure-time or occupational PA); however, it is of no use in estimating the duration of total PA.

Vietnam has 54 ethnic groups, among which the Kinh ethnic group accounted for more than 80% of the national population. The Kinh people are considered the most developed group, and most of them reside in the most advantaged regions in Vietnam. In our study, this group participated in PA less than the ethnic minority groups. Our study result is consistent with that in a study by Chang Ying [21] and another by Trinh et al. [22]. As ethnic minority people live in remote and mountainous areas, they are more likely to engaged in PA (e.g. walking up and down hills/mountains, bicycling, or doing farm work). Meanwhile, the Kinh ethnic group with better education are more likely to do mental jobs.

In our study, individuals aged 30–49 years were more likely to participate in PA. They dominated labor force; therefore, it is undoubted that they had the highest intensity of PA. As PA is highly associated with work, the working-age population is more likely to do PA. However, some authors such as Hallal et al. [18] revealed that older age was associated with reduced total PA. Considering each PA domain, some other authors found that older adults had higher leisure-time PA than children because the former had more leisure time, were more interested in doing PA for the sake of their health, and could afford leisure-time PA.

### Strengths and limitations

Our study had a number of strengths. The high response rates, the national samples and the multistage clustered random selection of samples help to ensure that the data were nationally representative. Another strength of this study was the large-scale sample selected from the whole country’s population. The GPAQ was used to identify the level of PA in the three domains and provide more information about the trends in the PA level of Vietnamese citizens. However, our study also encountered some limitations such as recall bias and inaccurate estimation which were inevitable due to self-report of PA levels. The level of PA was over-estimated due to recall bias, and the study respondents might give socially accepted answers other than those reflecting what they “really” thought (social desirability bias).

Previous studies advocated for assessment of total PA [36]. However, other also suggested that the sub-domain of PA (i.e., leisure PA or occupational PA) may have different impacts on health and should be considered separately [37]. This study analyzed secondary data from the national survey that used the GPAQ to collect PA-related information. The GPAQ only applies to the assessment of total PA, but not to each domain [38]. Future studies may need to analyze leisure-time and occupational PA separately in order to present a comprehensive picture of the level of PA in the general Vietnamese population.

## Conclusions

As PA domains may have different determinants and directions of association with SES, examining them in the studied population is of great importance. The study reported a negative association between SES and PA because most PA of the Vietnamese population was work-related; low SES groups came out as more active. Future studies should examine PA domains separately to provide practitioners with further recommendations on low/high SES populations in developing countries.

## Data Availability

The datasets used and/ or analyzed during the current study were from the STEPs 2015 survey which are available from General Department of Preventive Medicine, Ministry of Health, Vietnam upon contact dp@moh.gov.vn on reasonable request.

## Abbreviations

GPAQ: Global PA Questionnaire
GSO: General Statistics Office
METS: Metabolic Equivalent Tasks
MOH: Ministry of Health
PA: Physical activity
PCA: Principal component analysis
SES: Social-economic status
STEPS: Stepwise approach to Surveillance
VLSS: The Vietnam household living standard survey
